# Proteomic analysis of hepatic effects of okadaic acid in HepaRG human liver cells

**DOI:** 10.17179/excli2023-6458

**Published:** 2023-10-31

**Authors:** Leonie T.D. Wuerger, Greta Birkholz, Axel Oberemm, Holger Sieg, Albert Braeuning

**Affiliations:** 1 German Federal Institute for Risk Assessment, Department of Food Safety, Max-Dohrn-Str. 8-10, 10589 Berlin, Germany

**Keywords:** okadaic acid, HepaRG cells, liver proteome

## Abstract

The marine biotoxin okadaic acid (OA) is produced by dinoflagellates and enters the human food chain by accumulating in the fatty tissue of filter-feeding shellfish. Consumption of highly contaminated shellfish can lead to diarrheic shellfish poisoning. However, apart from the acute effects in the intestine, OA can also provoke toxic effects in the liver, as it is able to pass the intestinal barrier into the blood stream. However, molecular details of OA-induced hepatotoxicity are still insufficiently characterized, and especially at the proteomic level data are scarce. In this study, we used human HepaRG liver cells and exposed them to non-cytotoxic OA concentrations for 24 hours. Global changes in protein expression were analyzed using 2-dimensional gel electrophoresis in combination with mass-spectrometric protein identification. The results constitute the first proteomic analysis of OA effects in human liver cells and indicate, amongst others, that OA affects the energy homeostasis, induces oxidative stress, and induces cytoskeletal changes.

## Introduction

Okadaic acid (OA) is one of the main toxins to cause diarrheic shellfish poisoning (DSP). It is produced by dinoflagellates of the genus *dinophysis* and *prorocentrum* (EFSA, 2008[6]). These dinoflagellates probably produce the toxin to gain an advantage against other microalgae (Gong et al., 2021[10]). Under optimal conditions, OA-expressing dinoflagellates can explosively grow, leading to so-called harmful algae blooms. Due to climate change and the industrial waste of humans, the occurrence of harmful algae blooms has significantly increased in the last years, leading to an increase in OA occurrence (Van Dolah, 2000[35]). OA is very lipophilic, thereby accumulating in the fatty tissue of filter-feeding shellfish, through which they can also enter the human food chain. In humans, OA can cause DSP, which leads to severe gastrointestinal symptoms, like vomiting, diarrhea and stomach pain. Based on that, the European Union has implemented a limit of 160 OA equivalents/kg shellfish meat, based on the acute reference dose of 0.3 µg OA equivalents/kg body weight (FAO, 2004[7]). 

While the most extensively researched acute effects of OA intoxication are observed in the intestine, effects on other organs have been investigated and understood much less. However, it was shown that OA is distributed throughout the whole body *in vivo* and that it has a particularly long retention time in the liver (Matias et al., 1999[25]; Ito et al., 2002[16]). Furthermore, OA is able to inhibit several protein phosphatases, mainly protein phosphatase 1 and 2A (Bialojan and Takai, 1988[2]). Therefore, it has the potential to severely influence kinase- and phosphorylation-dependent signaling pathways in the liver. There is also evidence that OA acts as a potent tumor promotor in animals (Jiménez-Cárcamo et al., 2020[17]; Messner et al., 2006[26]) and it might be the cause of cancer cases reported in epidemiological studies (Cordier et al., 2000[3]; Lopez-Rodas et al., 2006[22]; Manerio et al., 2008[24]). OA was also found to be cytotoxic in several different cell lines, including liver cells (Ferron et al., 2014[8]; Fessard et al., 1996[9]; Le Hégarat et al., 2006[21]; Rubiolo et al., 2011[28]). The toxin is furthermore able to interfere with xenobiotic metabolism in the liver by inhibiting several enzymes of hepatic biotransformation (Vieira et al., 2013[36]; Wuerger et al., 2022[39]). Apart from that, the information on the effects of OA on the liver is very limited. 

In a comparative proteomics 2D-PAGE analysis of mouse liver, Wang et al. found 46 deregulated proteins involved in macromolecular metabolism, molecular chaperone/stress response, apoptosis, and cytoskeleton formation after chronic exposure to OA (Wang et al., 2021[37]). This study in mice is, to the best of our knowledge, the only available study describing changes to the liver proteome after OA exposure. The only other proteomic study with OA is reported in a publication from the same research group and describes proteomic alterations in mouse small intestine after a single oral dose of OA (Wang et al., 2012[38]). Technically, both studies were performed using gel-based 2-dimensional separation of proteins, followed by mass-spectrometric identification of deregulated proteins.

In order to close the existing knowledge gap regarding the effects of OA on human liver cells, we conducted a proteomic study of human HepaRG liver cells exposed to different concentrations of OA. HepaRG cells are able to differentiate into hepatocyte-like cells, which express a variety of liver-specific markers and xenobiotic-metabolizing enzymes at a level similar to primary human hepatocytes (Kanebratt and Andersson, 2008[19]; Tascher et al., 2019[32]). 2-dimensional gel electrophoresis and subsequent mass-spectrometric protein identification was chosen in order to generate data suitable for a direct comparison to the results from the two studies by Wang and co-workers (Wang et al., 2012[38], 2021[37]).

## Materials and Methods

### Chemicals

OA was purchased from Enzo Life Sciences GmbH (Loerrach, Germany). All other standard chemicals and materials were purchased either from Sigma-Aldrich (Taufkirchen, Germany) or Roth (Karlsruhe, Germany) in the highest available purity.

### Cell cultivation

HepaRG cells (Biopredic International, Saint-Grégoire, France) were cultivated at 37 °C for 14 days in William's E medium supplemented with 10 % fetal bovine serum (FBS), 5 μg/mL insulin (medium and both supplements from PAN-Biotech GmbH, Aidenbach, Germany), 50 μM hydrocortisone hemisuccinate (Sigma Aldrich, Taufkirchen, Germany), and with 100 U/mL penicillin and 100 μg/mL streptomycin (Capricorn Scientific, Ebsdorfergrund, Germany). After 14 days, the medium was supplemented with 1 % dimethyl sulfoxide (DMSO) for 2 days to start differentiation of the cells. After that, the DMSO content was increased to 1.7 % for another 12 days. The medium was then changed to serum-free assay medium (SFM), which was adapted from Klein et al. (2014[20]). SFM consisted of William's E medium without phenol red (PAN-Biotech GmbH, Aidenbach, Germany), supplemented with 100 U/mL penicillin and 100 μg/mL streptomycin, 2.5 μM hydrocortisone hemisuccinate, 10 ng/mL human hepatocyte growth factor (Biomol GmbH, Hamburg, Germany), 2 ng/mL mouse epidermal growth factor (Sigma Aldrich, Taufkirchen, Germany), and 0.5 % DMSO. Cells were incubated with 33 nM OA, 100 nM OA or the respective solvent control for 24 hours.

### Cell viability testing

Cell viability testing was conducted as previously reported (Wuerger et al., 2022[39]). In short, 9 × 10^3 ^cells/well were seeded in 96-well plates and cells were cultivated as described above. Cells were then incubated with different OA concentrations for 24 h. Cell viability was subsequently determined using the tetrazolium dye 3-(4,5-dimethylthiazol-2-yl)-2,5-diphenyltetrazolium bromide (MTT; Biomol GmbH, Hamburg, Germany).

### Protein extraction

The HepaRG cells were washed in ice-cold PBS and then scraped in PBS. The cells were then centrifuged (5 min, 2000 x g, 4 °C). For protein extraction from HepaRG cell pellets, RIPA lysis buffer (pH 7.5; 50 mM Tris-HCl, 150 mM NaCl, 2 µM EGTA, 0.1 % sodium dodecyl sulfate (SDS), and 0.5 % desoxycholic acid) containing 1:50 protease inhibitor (Complete Protease Inhibitor Cocktail Tablets, Roche, Mannheim, Germany) and 1 % Triton X-100 was used. The cells were lysed by rotating at 4 °C for 15 min, and homogenization was carried out using an ultrasonic homogenizer (Sonopuls HD 2070, BANDELIN electronic GmbH & Co. KG, Berlin, Germany, 25 % power, pulse 2). The homogenized lysates were then centrifuged at 4 °C and 13,200 x g for 30 min. The supernatant was collected and stored at -80 °C. The protein content in the resulting supernatants was determined using the Bradford assay according to the manual of the Bio-Rad protein assay (Bio-Rad Laboratories GmbH, Feldkirchen, Germany) against a bovine serum albumin standard curve.

### 2-dimensional gel electrophoresis

2-dimensional gel electrophoresis was carried out according to the protocol by Görg et al. (2000[11]). In brief, isoelectric focusing (IEF) was done using 24-cm IPG gel strips, which were loaded with 150 μg protein; gel running conditions are detailed in Görg et al. (2000[11]). After equilibration, SDS-polyacrylamide gel electrophoresis was performed with laboratory-made 12.5 % acrylamide gels. Subsequent to fixation in aqueous acetic acid/ethanol, gel staining was performed using Ruthenium II Tris solution (pH 7; 20 mM potassium tetrachloroaurate (III), 0.16 mM bathophenanthrolinedisulfonic acid disodium salt hydrate, 96 mM sodium ascorbate). The stained gels were scanned in a Molecular Imager Versadoc MP 4000 (Bio-Rad Laboratories GmbH, Feldkirchen, Germany), with an excitation wavelength at 450 nm and emission wavelength at 605 nm. Analysis of the images was carried out using ProteinMine software (Scimagix, San Mateo, CA, USA) with a minimal spot size of 8 and a sensitivity of 35. The generated data was then analyzed in R (R Core Team) using an internal script that uses the Wilcoxon Rank sum test to determine the differentially expressed protein spots (cutoffs: p ≤ 0.01 and │log_2_ ratio│ ≥ 0.5). Spots were included if detected in 2 out of 3 biological and in 2 out of 4 technical replicates. 

### Mass-spectrometric protein identification

The annotated protein spots were identified using MALDI mass spectrometry. The spots were cut from the gels and dehydrated using acetonitrile (Merck KGaA, Darmstadt, Germany). The dehydrated gel spots were then rehydrated for 30 minutes at 4 °C using a trypsin solution (0.425 µM trypsin (Roche, Mannheim, Germany) in 50 mM ammonium bicarbonate (pH 8, Sigma Aldrich, Taufkirchen, Germany)). After 30 minutes, the retaining solution was discarded and the gel pieces were put into a 50 mM ammonium bicarbonate solution (pH 8; Sigma Aldrich, Taufkirchen, Germany). The spots were digested over night at 37 °C. Then, trifluoroacetic acid (Merck KGaA, Darmstadt, Germany) was added to the spots to extract the peptides. Each spot was analyzed using an Ultraflex II MALDI-TOF/TOF mass spectrometer (Bruker Daltonics, Billerica, Massachusetts, USA). The corresponding proteins for each spot were then identified using MASCOT (Matrix Science, London, UK). Proteins were sorted into categories related to different cellular processes using the UniProt database (https://www.uniprot.org/2023).

## Results

**Figure 1:**
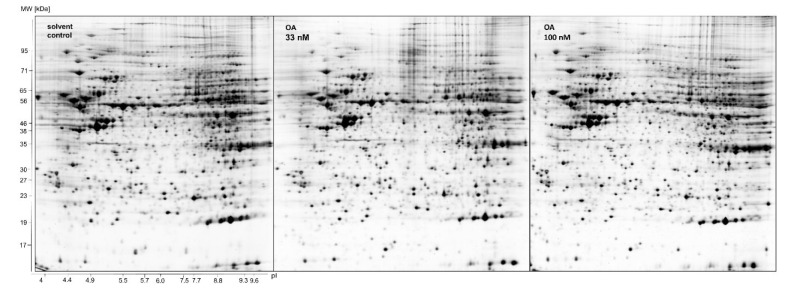
Representative gels of separated proteins from HepaRG cells incubated with 33 or 100 nM OA for 24 h. HepaRG cells were lysed, and proteins were extracted. Proteins were first separated using isoelectric focusing and then further using SDS-PAGE. Gels were stained using Ruthenium II Tris solution, n=3.

**Figure 2:**
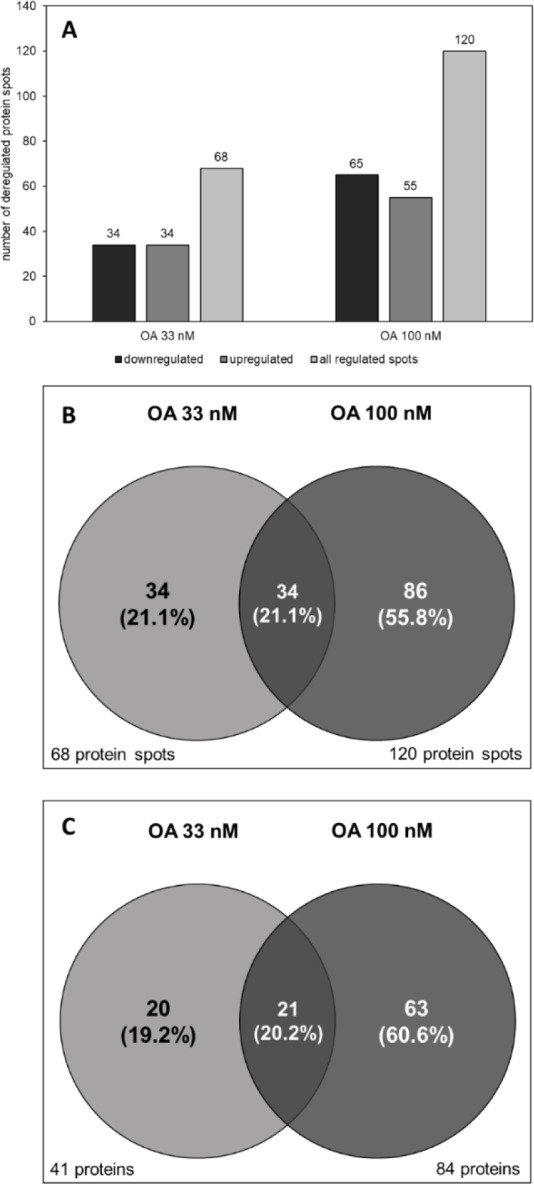
Deregulated protein spots and identified deregulated proteins in OA-treated HepaRG cells. Proteins were isolated from HepaRG cells incubated with 33 or 100 nM OA for 24 h. Proteins were then separated using 2D gel electrophoresis and deregulated protein spots were identified using the Wilcoxon Rank sum test (p ≤ 0.01 and │log_2_ ratio│≥ 0.5). (A) Total numbers of up- and downregulated protein spots. (B) Venn diagram visualization of the overlap of deregulated protein spots between the treatment groups, presented in absolute numbers and percentages. (C) The proteins corresponding to each spot were identified using MALDI-MS; the diagram shows the numbers and percentages of identified proteins in the treatment groups and their overlap.

To assess changes in the liver proteome after exposure to OA, we analyzed the proteome from differentiated HepaRG cells after exposure to non-toxic concentrations of OA using 2-dimensional gel electrophoresis. Non-toxic concentrations of OA were determined using the MTT assay. From the data shown in Supplementary Figure 1, 33 nM and 100 nM were selected as moderate and high non-toxic OA concentrations, respectively. After incubation with OA, 2D gels were run as described in the “Methods” section. Figure 1(Fig. 1) depicts representative gels of each treatment group. Gels were stained using Ruthenium II Tris solution. The spot patterns on the gels were then detected using the software ProteinMine and analyzed using the Wilcoxson rank sum test. Only spots that were detected in at least two of the three biological and in two of the four technical replicates were considered for the analysis. We were able to find 68 significantly deregulated protein spots in the 33 nM OA group (p ≤ 0.01 and |log_2_ ratio| ≥ 0.5), 34 of which were upregulated and 34 downregulated. In the 100 nM OA group we detected 65 downregulated protein spots and 55 upregulated spots, which adds up to a total of 120 deregulated spots (Figure 2A(Fig. 2)). 34 protein spots were overlapping between both groups (Figure 2B(Fig. 2)). The combination of a comparably strict p value and a moderate fold change cutoff was chosen to allow for the identification of only slightly deregulated proteins, while at the same time avoiding false-positive results. The individual proteins present in the deregulated protein spots were then identified using MALDI-MS. 41 proteins were identified for the 33 nM group (identification rate 60 %) and 84 proteins for the 100 nM group (identification rate 70 %), with 21 proteins that were found in both groups (Figure 2C(Fig. 2)). The most upregulated protein was peroxiredoxin 6, which was found in the 100 nM treatment group (Table 1(Tab. 1)). The 15 most upregulated proteins are listed in Table 1(Tab. 1). The most downregulated protein was isocitrate dehydrogenase (NADP) 1, which was found in the 33 nM treatment group (Table 2(Tab. 2)). The 15 most downregulated proteins are listed in Table 2(Tab. 2). The full list of deregulated proteins is contained in Supplementary Table 1. Please note that individual proteins might be present and detected as different spots on the gels, for example when a protein occurs in different post-translational modifications. Therefore, appearance of a certain protein in the lists for up- and downregulated proteins does not invalidate the data, but points towards a shift between protein isoforms. Moreover, changes detected by 2-DIGE/MALDI-MS might not be reflected by changes in the overall amount of a protein (i.e., the sum of all differently modified forms of it).

**Figure 3:**
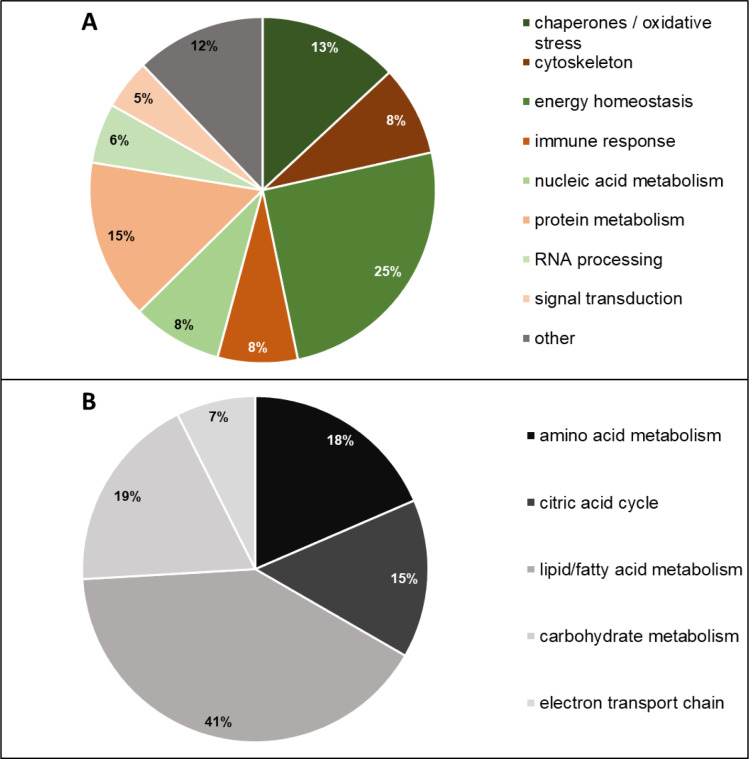
Grouping of identified OA-regulated proteins according to their cellular functions. (A) Distribution of the proteins between different cellular functions and processes according to the UniProt database. (B) Detailed functional grouping of the proteins related to energy homeostasis

The identified proteins were assigned to different cellular functions and processes using the UniProt database (Figure 3(Fig. 3)). It was observed that 25 % of all identified proteins were associated with energy homeostasis (for example, PDHB, GLUL and ETFA), 15 % were related to protein metabolism (for example, EEF2, EIF4H or UBQLN1), and 13 % were chaperones or proteins involved in oxidative stress-related processes (for example, HSP90B1, HSPA4 or PRDX6; Figure 3A(Fig. 3)). As one quarter of all deregulated proteins were connected with energy homeostasis, we further assigned them to the different pathways of the energy metabolism (Figure 3B(Fig. 3)). The largest group of the aforementioned proteins (41 %) take part in the metabolisms of lipids and/or fatty acids (for example ACAT1, APOE or HEXA), while another 19 % were also associated to carbohydrate metabolism (for example, PGM1, TALDO1, ALDOC) and another 18 % were proteins of the amino acid metabolism (for example, ACY1, GLUL or HIBCH). This indicates that both, lipid and carbohydrate metabolism, are substantially affected by OA in HepaRG cells.

## Discussion

Even though OA has mostly been recognized for its acute effects in the gut, but is able to pass the intestinal barrier and reach the liver, where it has a long retention time (Ito et al., 2002[16], Matias et al., 1999[25], Dietrich et al., 2019[5]). Nonetheless, only few hepatic effects of OA have been characterized so far. For example, a mechanism for the OA-dependent downregulation of drug metabolism-related enzymes in human liver cells has been recently deciphered (Wuerger et al., 2022[39], 2023[40]). Not much, however, is known about the overall proteomic response of liver cells to OA, and only a single proteomic study with mouse liver after chronic exposure to OA has been published in the past (Wang et al., 2021[37]). Additional proteomic information comes from a study with mouse intestine (Wang et al., 2012[38]), and a limited proteomic data set has also been generated from isolated lipid rafts of a human neuroblastoma cell line (Opsahl et al., 2013[27]). We aimed at closing the knowledge gap regarding hepatic proteomic effects of OA by using a well-established *in vitro *model of human liver, HepaRG cells, in combination with gel-based protein separation and mass-spectrometric protein identification. This was done in order to generate a proteomic data set allowing the comparison with the aforementioned published data based on a technically comparable approach. Hepa-RG cells were incubated with non-toxic doses of OA, to detect OA-specific alterations and to avoid the occurrence of unspecific cytotoxic responses of the cells. Therefore, the present study is the first to provide an unbiased proteomic analysis of OA-induced alterations in human cells, and the first proteomic study of hepatic OA effects conducted with human cells. In our study, a total number of 104 proteins were identified, as compared to 46 proteins in mouse liver and 58 proteins in mouse intestine, respectively, in previous studies (Wang et al., 2012[38], 2021[37]). Thus, our results constitute a substantial improvement of knowledge regarding the cellular effects of OA at the protein level.

Supplementary Table 1 lists the deregulated proteins identified in our analysis, along with information whether the respective proteins (i.e., their direct murine orthologs, or similar isoforms) have been identified as OA-regulated in the two aforementioned publications by Wang and co-workers. In fact, congruence of 34 entries (28 %) from our Hepa-RG-derived protein list with proteins previously identified as being affected by OA in mouse liver or intestine was noted (Supplementary Table 1). This high level of congruence, despite the species difference and the differences in study design and analytical workflow, substantiates the validity of the results obtained in our study.

One quarter of the OA-regulated proteins identified in this study were functionally associated with energy homeostasis, and most of them are part of pathways related to lipid or fatty acid metabolism. The liver is one of the most important organs for energy homeostasis in the human body (Rui, 2014[29]). It is the central organ for fatty acid metabolism. This includes, among other processes, the *de novo* synthesis and also the elimination of fatty acids (Alves-Bezerra and Cohen, 2017[1]). It has been shown that OA is able to promote lipolysis, thereby interfering with fatty acid metabolism (He et al., 2006[13]). The liver furthermore plays a central role in glucose metabolism, including glycogenesis, glycogenolysis, glycolysis and gluconeogenesis (Han et al., 2016[12]). Previous data indicate that OA is able to interfere with glucose metabolism in hepatocytes by increasing gluconeogenesis and glucose output (Louzao et al., 2005[23]). Thus, the identification of a high percentage of OA-deregulated proteins as associated with fat and energy metabolism is in line with previous data. Only one core component of glycolysis (ALDOC) was found affected by OA in our analysis, while a number of proteins related to the tricarboxylic acid cycle (ACO2, IDH1, PDHA1, PDHB) were also identified as altered in OA-treated HepaRG cells.

Exposure to OA is able to induce inflammatory responses. For example, OA is able to activate the expression and release of different proinflammatory cytokines *in vitro* (Del Campo et al., 2017[4]; Suuronen et al., 2006[31]; Tchivelekete et al., 2022[33]; Wuerger et al., 2023[40]). An increase of IL-1β caused by OA was furthermore detected in rat brains *in vivo* (Kamat et al., 2012[18]). OA is furthermore a known inducer of reactive oxygen species (ROS) (Schmidt et al., 1995[30]; Túnez et al., 2003[34]), and therefore a cellular ROS-related response following OA exposure is to be expected. 13 % of the identified proteins in our analysis were chaperones or part of the cellular response to oxidative stress. Similar results were also obtained by Wang et al., where a comparable percentage of the identified proteins in mouse liver was related to oxidative stress and/or chaperone function (Wang et al., 2021[37]). 

Another noteworthy aspect of our results is that 8 % of the identified proteins were part of the cytoskeleton. Wang et al. also found an effect of OA on the cytoskeleton in mouse liver, with 13 % of their identified proteins being part of it (Wang et al., 2021[37]). In the second study by Wang et al., the authors also found a large number of the regulated proteins in mouse intestine to be part of the cytoskeleton (Wang et al., 2012[38]). The cytoskeleton is, among others, responsible for the overall integrity of the cell, cell adhesion, and cell-cell contacts (Huber et al., 2013[15]). One of the known main targets of OA is the cytoskeleton. This leads, for example, to a disruption of tight junctions or actin filaments (Dietrich et al., 2019[5]; Opsahl et al., 2013[27]; Huang et al., 2023[14]). Our data provide additional evidence for effects of OA on the cytoskeleton and thus broaden our knowledge on this aspect of the molecular toxicity of OA.

In conclusion, our data confirm and substantially extend our knowledge regarding proteomic effects of okadaic acid in mammalian cells and, for the first time, provide insight into hepatocellular proteomic effects of OA in human cells.

## Declaration

### Author contributions 

LW 1,2,3,4

GB 1,2

AO 1,2

HS 3,4

AB 3,4,5

1: Performed experiments

2: Data evaluation

3: Writing the manuscript

4: Project planning

5: Supervision and funding

### Declaration of competing interest

We declare that there is no competing interest.

### Acknowledgment

We would like to thank Christine Meckert for technical assistance. This project was supported by the German Federal Institute for Risk Assessment, Germany (project 1322-824).

## Supplementary Material

Supplementary information

## Figures and Tables

**Table 1:**
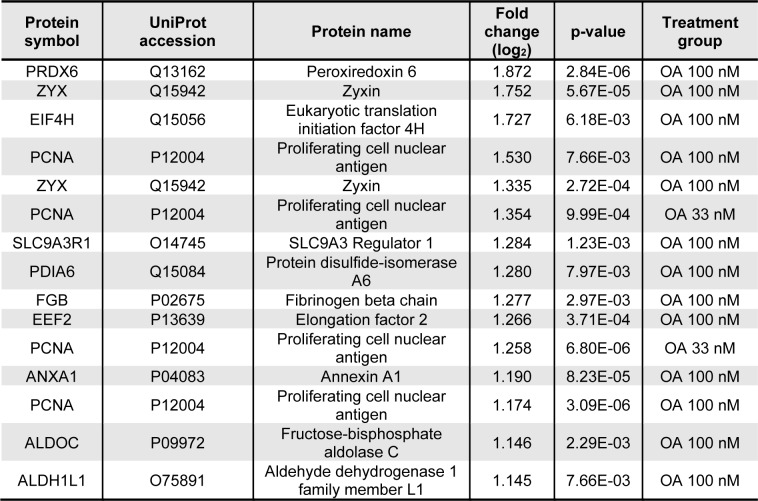
The 15 most prominently upregulated proteins in all samples. Proteins were isolated from HepaRG cells incubated with 33 or 100 nM OA for 24 h. Proteins were then separated using 2D gel electrophoresis and deregulated proteins were identified using MALDI-MS.

**Table 2:**
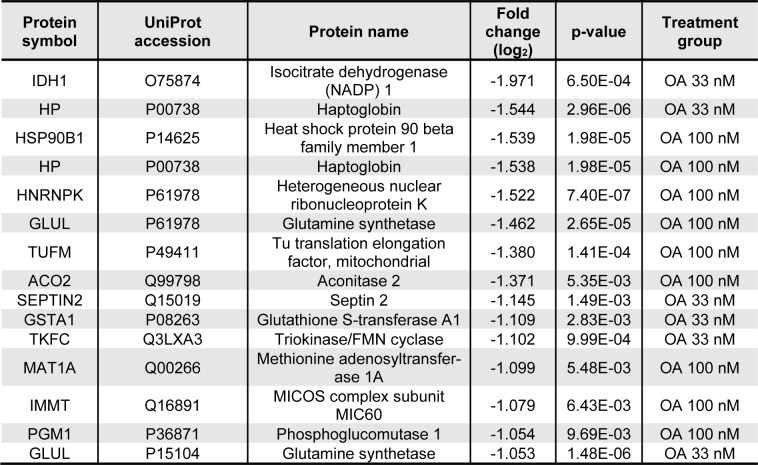
The 15 most prominently downregulated proteins in all samples. Proteins were isolated from HepaRG cells incubated with 33 or 100 nM OA for 24 h. Proteins were then separated using 2D gel electrophoresis and deregulated proteins were identified using MALDI-MS.
