# Supplementary material for: Proteomic analysis of hepatic effects of okadaic acid in HepaRG human liver cells
Source: EXCLI J. 2023 Oct 31;22:1135–45. doi: 10.17179/excli2023-6458 (PMC10694344; doi:10.17179/excli2023-6458)
Supplement: Supplementary information [file EXCLI-22-1135-s-001.pdf]

EXCLI Journal 2023;22:1135-1145 – ISSN 1611-2156
Received: August 10, 2023, accepted: October 18, 2023, published: October 31, 2023

Supplementary information to:

Original article:

PROTEOMIC ANALYSIS OF HEPATIC EFFECTS OF OKADAIC ACID IN HEPARG HUMAN LIVER CELLS

Leonie T.D. Wuerger, Greta Birkholz, Axel Oberemm, Holger Sieg*, Albert Braeuning[a]

German Federal Institute for Risk Assessment, Department of Food Safety, Max-Dohrn-Str. 8-10, 10589 Berlin, Germany

* **Corresponding author:** Holger Sieg, German Federal Institute for Risk Assessment, Department of Food Safety, Max-Dohrn-Str. 8-10, 10589 Berlin, Germany; E-mail: holger.sieg@bfr.bund.de

https://dx.doi.org/10.17179/excli2023-6458

This is an Open Access article distributed under the terms of the Creative Commons Attribution License (http://creativecommons.org/licenses/by/4.0/).

EXCLI Journal 2023;22:1135-1145 – ISSN 1611-2156
Received: August 10, 2023, accepted: October 18, 2023, published: October 31, 2023

Supplementary Table 1: List of all regulated proteins in both treatment groups. Proteins were isolated from HepaRG cells incubated with 33 or 100 nM OA for 24 h. Proteins were then separated using 2D gel electrophoresis and deregulated proteins were identified using MALDI-MS. Deregulation of individual proteins by OA in mouse intestine (Wang et al., 2012) and in mouse liver (Wang et al., 2021) is presented in the row ”comparison with published data”: 1, mouse ortholog of detected protein deregulated in Wang et al., 2012; (1), similar protein isoform deregulated in Wang et al., 2012; 2, mouse ortholog of detected protein deregulated in Wang et al., 2021; (2), similar protein isoform deregulated in Wang et al., 2021.

			OA 33 nM		OA 100 nM		
**Protein symbol**	**UniProt Accession number**	**Protein name**	**Fold change (log_2)**	**p-value**	**Fold change (log_2)**	**p-value**	**Comparison with published data**
**ABHD14B**	Q96IU4	Protein ABHD14B			-0.657	2.84E-06	
**ACAT1**	P24752	Acetyl-CoA acetyltransferase 1	0.623	6.58E-03			
**ACO2**	Q99798	Aconitase 2			-0.606	7.38E-03	
**ACO2**	Q99798	Aconitase 2			-1.371	5.35E-03	
**ACOT1**	Q86TX2	Acyl-coenzyme A thioesterase 1	-0.507	5.95E-03			
**ACY1**	Q03154	Aminoacylase 1			-0.543	1.08E-03	1
**ACY1**	Q03154	Aminoacylase 1			-0.556	1.12E-04	1
**AHSA1**	O95433	Activator of 90 kDa heat shock protein ATPase homolog 1	1.088	2.06E-03	0.992	1.39E-04	
**AK2**	P54819	Adenylate kinase 2			-0.939	4.14E-03	
**AKR1A1**	P14550	Aldo-keto reductase family 1 member A1			-0.632	7.31E-03	
**ALDH1L1**	O75891	Aldehyde dehydrogenase 1 family member L1			1.145	7.66E-03	1
**ALDH2**	P05091	Aldehyde dehydrogenase 2			-0.740	8.33E-03	(1)
**ALDOC**	P09972	Fructose-bisphosphate aldolase C			1.147	2.29E-03	
**ANXA1**	P04083	Annexin A1			0.907	1.98E-05	(2)
**ANXA1**	P04083	Annexin A1			1.191	8.23E-05	(2)
**ANXA8**	P13928	Annexin A8			-0.680	4.14E-03	(2)
**APOE**	P02649	Apolipoprotein E			0.898	2.75E-04	
**ARFIP1**	P53367	Arfaptin-1			-0.538	7.99E-03	
**ARG1**	P05089	Arginase 1	-1.011	1.48E-06			(1)
**BAG2**	O95816	BAG family molecular chaperone regulator 2			0.515	9.58E-03	
**CAVIN1**	Q6NZI2	Caveolae associated protein 1			-0.732	2.75E-04	
**CCT5**	P48643	T-complex protein 1 subunit epsilon	0.601	1.56E-03	0.837	6.56E-03	(1)
**CES1**	P23141	Liver carboxylesterase 1	-0.841	8.65E-03			(2)
**CMPK1**	P30085	UMP-CMP kinase			-0.517	8.33E-03	
**CPOX**	P36551	Oxygen-dependent coproporphyrinogen-III oxidase			-0.714	4.29E-03	
**CRYL1**	Q9Y2S2	Lambda-crystallin homolog	-0.639	2.96E-03			
**CTSB**	P07858	Cathepsin B			-0.930	1.04E-03	
**CTSD**	P07339	Cathepsin D			-0.698	8.13E-03	
**CTSD**	P07339	Cathepsin D			-0.703	9.65E-03	
**DARS1**	P14868	Aspartate--tRNA ligase			-0.860	2.71E-03	

EXCLI Journal 2023;22:1135-1145 – ISSN 1611-2156
Received: August 10, 2023, accepted: October 18, 2023, published: October 31, 2023

**DNPH1**	O43598	2'-Deoxynucleoside 5'-phosphate N-hydrolase 1			-0.660	7.40E-04	
**DPYSL2**	Q16555	Dihydropyrimidinase-related protein 2			-1.026	4.96E-05	
**DYNC1I2**	Q13409	Dynein cytoplasmic 1 intermediate chain 2			-0.658	6.99E-03	
**ECHDC3**	Q96DC8	Enoyl-CoA hydratase domain containing protein 3			-0.545	3.94E-04	
**ECHS1**	P30084	Enoyl-CoA hydratase			-0.645	2.10E-04	
**EEF1D**	P29692	Elongation factor 1-delta	0.574	2.32E-03	0.674	2.74E-04	(1)
**EEF2**	P13639	Elongation factor 2			1.266	3.71E-04	1
**EEF2**	P13639	Elongation factor 2			0.583	5.46E-04	1
**EIF1AX**	P47813	Eukaryotic translation initiation factor 1A, X-chromosomal	0.529	4.00E-03			(1)
**EIF4A3**	P38919	Eukaryotic initiation factor 4A-III			0.830	1.13E-03	(1)
**EIF4H**	Q15056	Eukaryotic translation initiation factor 4H			1.728	6.18E-03	(1)
**ENO1**	P06733	Alpha-enolase	1.069	1.52E-03			
**ERLIN2**	O94905	Erlin-2			0.840	7.01E-03	
**ETFA**	P13804	Electron transfer flavo-protein subunit alpha			-0.675	9.49E-03	
**EZR**	P15311	Ezrin	0.569	1.43E-04	1.056	3.57E-04	
**EZR**	P15311	Ezrin	0.654	6.57E-03	0.675	9.79E-03	
**EZR**	P15311	Ezrin	0.746	3.71E-04	1.094	2.84E-06	
**EZR**	P15311	Ezrin			1.090	3.11E-05	
**FAH**	P16930	Fumarylacetoacetase			-0.592	2.80E-03	
**FAHD1**	Q6P587	Acylpyruvase FAHD1	-0.647	3.33E-03			
**FGA**	P02671	Fibrinogen alpha chain			0.828	4.10E-03	
**FGB**	P02675	Fibrinogen beta chain			1.278	2.97E-03	
**FGG**	P02679	Fibrinogen gamma chain	-0.563		-0.619	1.66E-03	
**GAA**	P10253	Lysosomal alpha-glucosidase			-0.640	1.38E-03	
**GARS1**	P41250	Glycine--tRNA ligase			0.845	1.13E-03	
**GATD3**	P0DPI2	Glutamine amidotrans-ferase-like class 1 domain-containing protein 3			-0.557	3.74E-04	
**GLUL**	P15104	Glutamine synthetase	-1.054	1.48E-06	-1.462	2.65E-05	
**GNMT**	Q14749	Glycine N-methyltrans-ferase	-0.907	4.84E-03			
**GRB2**	P62993	Growth factor receptor bound protein 2			0.884	3.77E-03	
**GSTA1**	P08263	Glutathione S-transfer-ase A1	-1.109	2.83E-03			
**HEBP1**	Q9NRV9	Heme binding protein 1			-0.511	9.64E-05	
**HEXA**	P06865	Beta-hexosaminidase subunit alpha			-0.568	1.86E-03	
**HIBCH**	Q6NVY1	3-Hydroxyisobutyryl-CoA hydrolase	-1.043	5.99E-03			
**HIBCH**	Q6NVY1	3-Hydroxyisobutyryl-CoA hydrolase	-0.522	3.60E-04			
**HNRNPF**	P52597	Heterogeneous nuclear ribonucleoprotein F	-0.728	6.79E-04	-0.756	4.90E-03	1

EXCLI Journal 2023;22:1135-1145 – ISSN 1611-2156
Received: August 10, 2023, accepted: October 18, 2023, published: October 31, 2023

**HNRNPK**	P31943	Heterogeneous nuclear ribonucleoprotein K	-0.877	7.40E-07	-1.523	7.40E-07	(1)
**HNRNPK**	P31943	Heterogeneous nuclear ribonucleoprotein K	-0.554	4.51E-03	-0.800	2.55E-04	(1)
**HP**	P00738	Haptoglobin	-1.544	2.96E-06	-1.539	1.98E-05	
**HSP90B1**	P14625	Heat shock protein 90 beta family member 1	-0.844	4.37E-03	-1.539	1.98E-05	1, (2)
**HSPA1A**	P0DMV8	Heat shock 70 kDa protein 1A	1.108	9.76E-03	0.650	9.61E-06	(1), (2)
**HSPA4**	P34932	Heat shock 70 kDa protein 4			-0.796	2.18E-03	(1), (2)
**HSPA8**	P11142	Heat shock cognate 71 kDa protein			-0.833	2.76E-03	(1), (2)
**HSPB1**	P04792	Heat shock protein beta-1			-0.662	1.78E-05	(1), (2)
**IDH1**	O75874	Isocitrate dehydrogen-ase (NADP) 1	-1.972	6.50E-04	-0.950	8.33E-03	
**IMMT**	Q16891	MICOS complex subunit MIC60			-1.079	6.43E-03	
**KRT8**	P05787	Keratin 8			1.072	7.14E-03	1, (2)
**KRT18**	P05783	Keratin 18			0.738	5.56E-03	(1), (2)
**LAP3**	P28838	Cytosol aminopeptidase			0.669	2.32E-03	
**LMNB2**	Q03252	Lamin B2	-0.621	8.41E-03			
**MAPRE1**	Q15691	Microtubule associated protein RP/EB family member 1			0.628	1.18E-03	
**MAT1A**	Q00266	Methionine adenosyl-transferase 1A			-1.100	5.48E-03	2
**MCCC2**	Q9HCC0	Methylcrotonoyl-CoA carboxylase beta chain			0.600	4.96E-04	
**NANS**	Q9NR45	Sialic acid synthase	0.778	8.27E-03			
**PCNA**	P12004	Proliferating cell nuclear antigen	1.354	9.99E-04	1.531	7.66E-03	
**PCNA**	P12004	Proliferating cell nuclear antigen	1.259	6.80E-06	1.174	3.09E-06	
**PDHA1**	P08559	Pyruvate dehydro-genase E1 subunit al-pha 1	-0.939	7.97E-04			
**PDHB**	P11177	Pyruvate dehydro-genase E1 subunit beta			-0.575	1.28E-04	
**PDIA6**	Q15084	Protein disulfide-isomer-ase A6			1.280	7.97E-03	1, 2
**PGM1**	P36871	Phosphoglucomutase 1	-0,948	8.30E-03	-1.054	9.69E-03	1
**PNP**	P00491	Purine nucleoside phosphorylase			0.851	5.76E-03	1
**PNPT1**	Q8TCS8	Polyribonucleotide nu-cleotidyltransferase 1			0.959	3.00E-03	
**PPIA**	P62937	Peptidyl-prolyl cis-trans isomerase A			-0.594	7.49E-03	
**PRDX4**	Q13162	Peroxiredoxin 4			-1.014	1.98E-05	(1), (2)
**PRDX6**	P30041	Peroxiredoxin 6	-0.601	1.28E-04	-0.655	1.37E-03	(1), (2)
**PRDX6**	P30041	Peroxiredoxin 6	0.866	7.48E-04	1.872	2.84E-06	(1), (2)
**RAN**	P62826	GTP-binding nuclear protein RAN	-0.502	5.64E-03			
**RBM8A**	Q9Y5S9	RNA-binding protein 8A			-0.861	1.74E-03	
**RCN1**	Q15293	Reticulocalbin 1	0.867	1.02E-03	0.895	2.30E-04	
**SELENBP1**	Q13228	Methanethiol oxidase	-0.607	5.24E-03			
**SEPTIN2**	Q15019	Septin 2	-1.145	1.49E-03	-0.651	5.08E-03	
**SERPINA1**	P01009	Serpin A1	1.093	8.25E-03			
**SERPINB9**	P50453	Serpin B9			0.845	1.42E-04	

EXCLI Journal 2023;22:1135-1145 – ISSN 1611-2156
Received: August 10, 2023, accepted: October 18, 2023, published: October 31, 2023

**SFN**	P31947	14-3-3 protein sigma	1.108	9.76E-03			
**SLC9A3R1**	O14745	SLC9A3 Regulator 1	0.903	3.26E-03	1.284	1.23E-03	
**SLC9A3R1**	O14745	SLC9A3 Regulator 1			0.559	1.28E-04	
**SND1**	Q7KZF4	Staphylococcal nuclease domain-containing protein 1	-0.693	7.32E-03	1.012	2.29E-03	
**SRI**	P30626	Sorcin			-0.686	2.84E-06	
**TALDO1**	P37837	Transaldolase 1			-0.810	5.30E-04	
**TBCB**	Q99426	Tubulin folding cofactor B			0.691	3.58E-03	
**TCP1**	P17987	T-complex protein 1 subunit alpha	-0.592	5.48E-04			(1)
**TKFC**	Q3LXA3	Triokinase/FMN cyclase	-1.103	9.99E-04			
**TPT1**	P13693	Translationally controlled tumor protein	0.505	5.53E-04	0.503	1.40E-03	
**TST**	Q16762	Thiosulfate sulfurtransferase			-0.585	1.29E-03	
**TUFM**	P49411	Tu translation elongation factor, mitochondrial			-1.381	1.41E-04	
**TUFM**	P49411	Tu translation elongation factor, mitochondrial			-0.531	2.93E-03	
**UBQLN1**	Q9UMX0	Ubiquilin 1			0.867	7.91E-03	
**UROD**	P06132	Uroporphyrinogen decarboxylase			-0.664	6.99E-03	
**ZYX**	Q15942	Zyxin	1.044	1.49E-03	1.752	5.67E-05	
**ZYX**	Q15942	Zyxin			1.336	2.72E-04	

EXCLI Journal 2023;22:1135-1145 – ISSN 1611-2156
Received: August 10, 2023, accepted: October 18, 2023, published: October 31, 2023

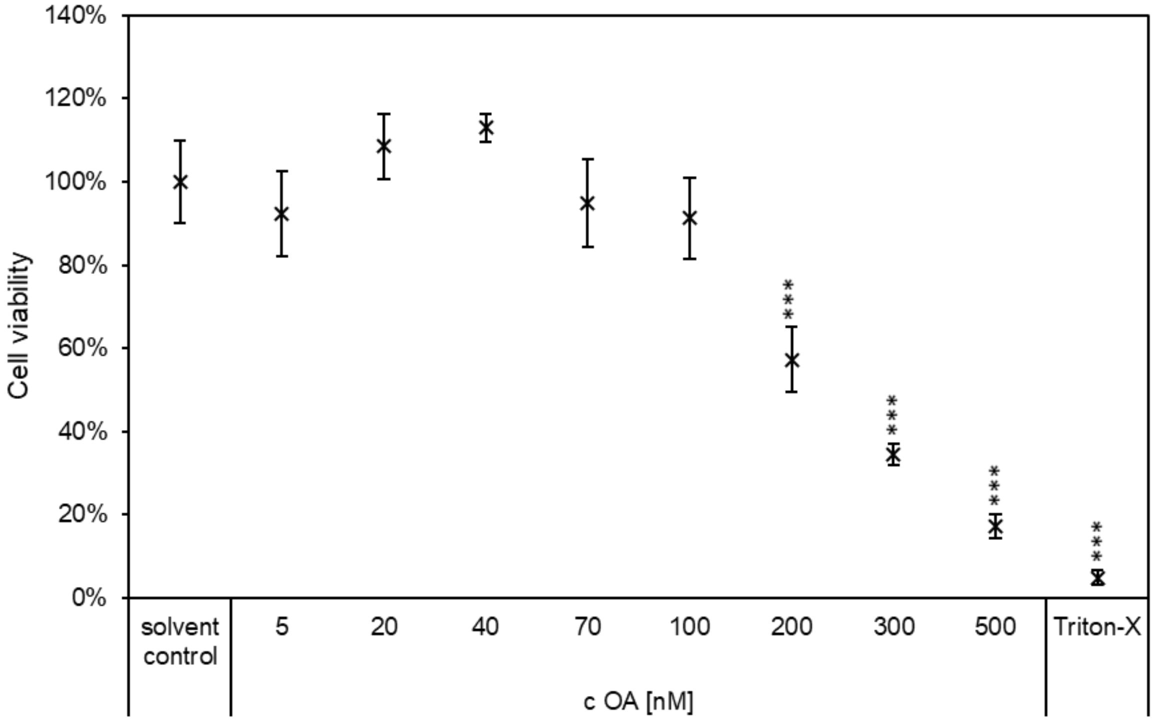

Supplementary Figure 1: MTT-assay of selected OA concentrations on HepaRG cells. Cells were seeded in 96-well plates and grown for 14 days, after which they were differentiated for another 14 days. They were incubated for 24 h with different OA concentrations. The absorption at 570 nm was measured and directly compared to the viability. Each sample was then compared to an untreated control. Based on the MTT assay, 100 nM and 33 nM OA were picked as non-toxic concentrations. Statistical analysis was performed using t-test (*p < 0.05; **p < 0.01; ***p < 0.001).
